# Sensory Aphasia After Cerebral Venous Sinus Thrombosis: Quantitative Evaluation of the Recovery Process of Language Function Using Tractography

**DOI:** 10.7759/cureus.80628

**Published:** 2025-03-15

**Authors:** Tsukasa Koike, Takahiro Tsuchiya, Atsumi Takenobu, Akira Teraoka

**Affiliations:** 1 Department of Neurosurgery, Teraoka Memorial Hospital, Fukuyama, JPN

**Keywords:** aphasia, arcuate fasciculus, arterial spin labeling (asl), cerebral venous sinus thrombosis (cvst), fractional anisotropy (fa), fsl, inferior fronto-occipital fasciculus, mrtrix, tractography, xtract

## Abstract

Cerebral venous sinus thrombosis (CVST) is a rare condition with high mortality that requires careful imaging evaluation. While arterial spin labeling (ASL) proves valuable for CVST assessment and diffusion tensor imaging (DTI) effectively evaluates language function in stroke, no studies have combined these techniques to assess the progression of higher brain dysfunction in CVST. A 59-year-old man presented with severe sensory aphasia. Magnetic resonance venography (MRV) showed left sigmoid sinus disruption with decreased proximal signal intensity, while conventional MRI showed no ischemic findings. The patient underwent sequential imaging with ASL and DTI using a 1.5 Tesla MRI system (SIGNA Explorer 1.5T, GE Healthcare Japan, Tokyo, Japan). The sensory aphasia was resolved by day two, coinciding with improvement in MRV and normalization of ASL findings. DTI tractography, analyzed using XTRACT auto-segmentation, showed changes in fractional anisotropy (FA) values of language-related white matter tracts that paralleled symptom improvement. Recovery of Chinese character reading and writing corresponded with changes in FA values in the inferior fronto-occipital and inferior longitudinal bundles, reflecting the resolution of venous congestion. This case demonstrates the complementary value of ASL and DTI in monitoring recovery from CVST. While ASL showed increased cerebral blood flow during the acute phase, DTI provided objective measures of functional recovery through changes in FA values in language-related white matter tracts. This combined imaging approach offers a promising method to assess both perfusion changes and functional recovery in CVST with associated higher brain dysfunction.

## Introduction

Cerebral venous sinus thrombosis (CVST) is a rare but severe form of stroke, accounting for 0.5-2% of all stroke cases, with a high fatality rate of 30-50% [1,2]. The primary diagnostic methods for CVST include magnetic resonance venography (MRV) and computed tomography venography (CTV), which detect thrombotic occlusion in venous sinuses [3]. However, beyond diagnosis, assessing functional recovery remains a challenge, particularly in cases involving higher brain dysfunction. Venous congestion in CVST can lead to reversible or permanent impairments in cognitive and language function, as blood flow disturbances may disrupt critical white matter tracts involved in neural processing. While arterial strokes affecting Wernicke’s area are more commonly associated with sensory aphasia, venous infarction and congestion in the posterior temporal lobe have also been implicated in transient language dysfunction. Munteanu et al. reported that approximately 20% of CVST patients experience language deficits, including sensory aphasia, with thrombosis of the transverse and sigmoid sinuses being the most common sites [4]. This suggests that venous congestion in the dominant hemisphere may disrupt language networks, leading to transient aphasia even in the absence of infarction. Traditional MRI sequences do not fully capture these functional changes, necessitating advanced imaging techniques. Arterial spin labeling (ASL) is a non-invasive perfusion imaging method that can quantitatively assess cerebral blood flow (CBF) without the need for contrast agents or radiotracers. Previous studies have demonstrated its utility in tracking the clinical course of CVST, particularly in detecting perfusion abnormalities during recovery [5]. Meanwhile, diffusion tensor imaging (DTI) has been widely used to assess white matter integrity and is particularly useful for evaluating language function in stroke patients [6-8]. Since fractional anisotropy (FA) values reflect microstructural white matter changes, tracking FA over time can provide insights into the recovery of language function and cognitive integrity following CVST. In this report, we utilize ASL to evaluate perfusion recovery in CVST and DTI-based FA analysis to assess white matter remodeling, allowing for a comprehensive assessment of functional recovery in a patient with CVST-related higher brain dysfunction.

## Case presentation

A 59-year-old right-handed man with no significant medical history was found uttering incomprehensible words, prompting a neighbor to call an ambulance. He was transported to our hospital, where an initial neurological assessment revealed no signs of paralysis, automatisms, or decreased consciousness. He exhibited difficulty processing both auditory and textual stimuli. His speech was fluent but characterized by a mixture of meaningful words, such as "China" and "war," interspersed with incomprehensible utterances. Additionally, his speech intonation resembled that of a non-native speaker. He was excessively talkative and displayed unilateral discourse patterns, rendering effective communication impossible. Based on these findings, language automatism was considered unlikely. A formal language function evaluation was conducted in collaboration with a speech therapist. Electroencephalography showed no epileptiform abnormalities, including spikes or spike-and-wave complexes indicative of temporal lobe epilepsy. To assess potential coagulation abnormalities, laboratory testing included protein C, protein S, and anti-cardiolipin antibody IgG, all of which were within normal limits. However, D-dimer was slightly elevated at 2.0 µg/ml, suggesting a possible hypercoagulable state. The patient had no history of chronic illnesses and was not taking any medications. His family history was unremarkable, except for his father, who had suffered a cerebral hemorrhage in late adulthood. He had a 28-year history of smoking 20 cigarettes per day but did not consume alcohol. Socially, he was unemployed and single.

**Figure 1:**
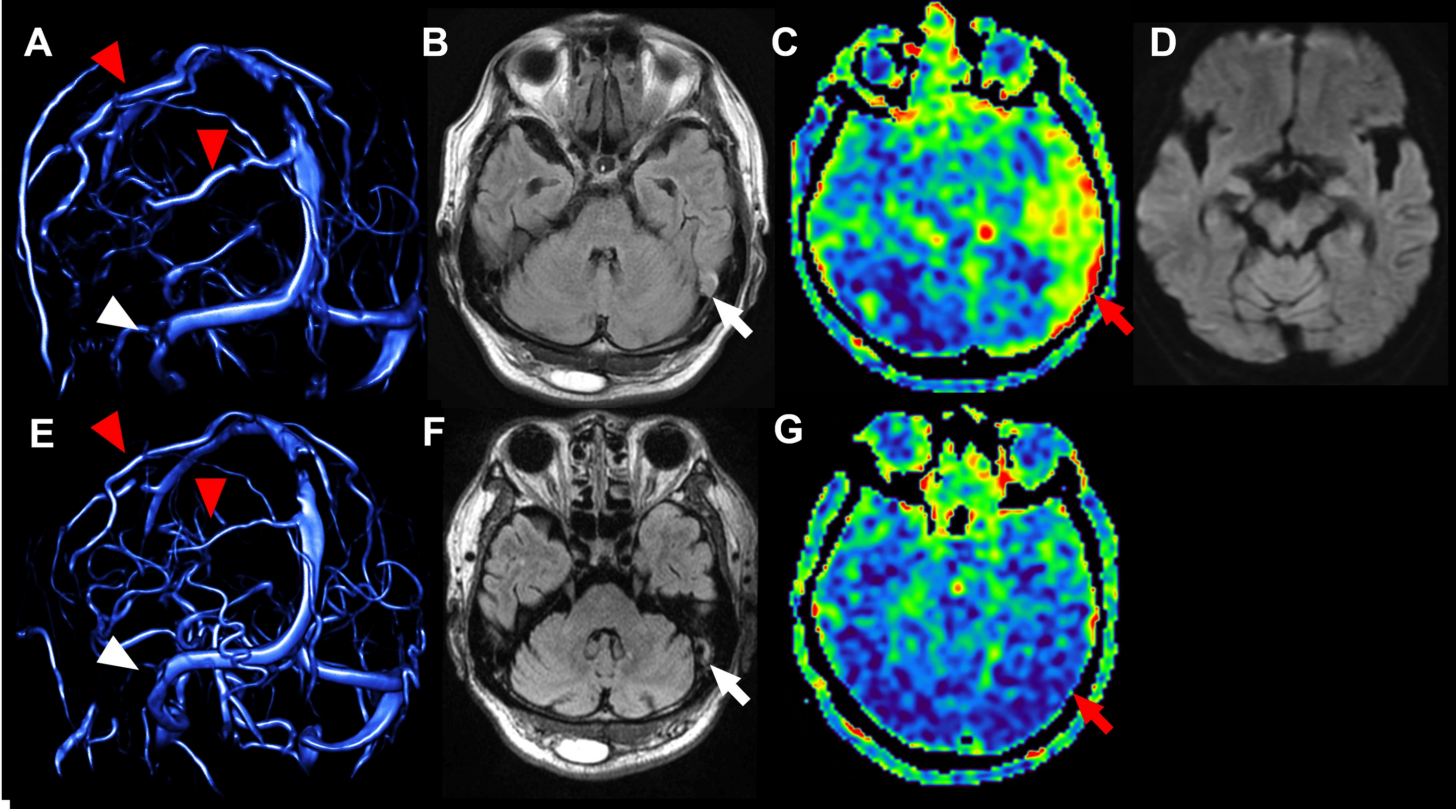
The transitions in the occlusion site of venous sinus and cerebral blood flow. (A) Magnetic resonance venography (MRV) on admission (day 0). The white arrowhead indicates the occluded portion of the left sigmoid sinus. The red arrowheads indicate dilated cortical vein. (B) Fluid-attenuated inversion recovery (FLAIR) on day 0. The left sigmoid sinus was nearly occluded. No significant differences in signal values were observed in the bilateral temporal lobe. (C) Arterial spin labeling (ASL) of day 0. The red arrow indicates increased cerebral blood flow (CBF) in the left temporal lobe. (D) The diffusion-weighted image of day 0 showed no ischemic or hemorrhagic lesion in the temporal lobe. (E) MRV on day two. The white arrowhead indicates the recanalized left sigmoid sinus. The red arrowheads indicate cortical veins that no longer demonstrate lateral asymmetry. (F) FLAIR on day two. The thrombus in the left sigmoid sinus has shrunk, and the stenosis has improved slightly. No laterality of signal intensity was observed in the bilateral temporal lobe. (G) The red arrow indicates that CBF in the left temporal lobe changed symmetrically.

A brain MRI showed no ischemic or hemorrhagic lesions, but MRV revealed a disruption of the left sigmoid sinus with reduced proximal signal intensity. Additionally, parietal cortical veins in the left hemisphere were dilated compared to the right hemisphere. Fluid-attenuated inversion recovery (FLAIR) imaging demonstrated near-total occlusion of the left sigmoid sinus due to a thrombus. No evidence of malformations or mass lesions in the medial temporal lobe, including mesial temporal sclerosis, was observed. ASL indicated increased CBF in the left temporal lobe compared to the right, suggesting hemodynamic compensation (Figure 1).

**Figure 2:**
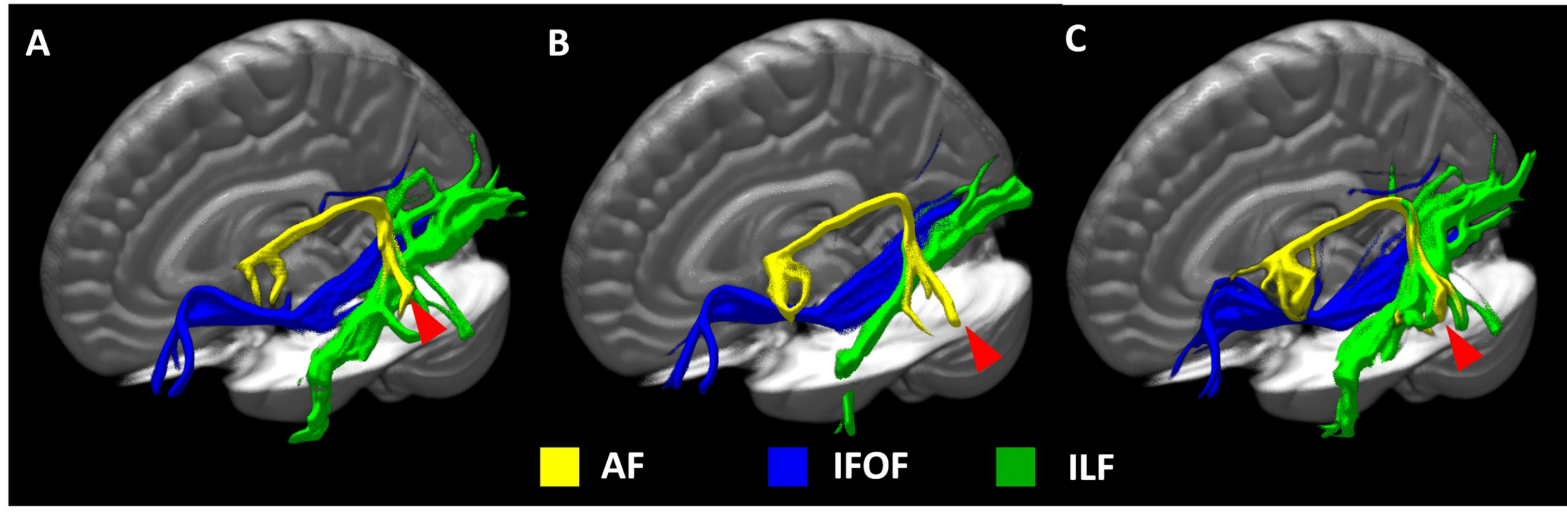
Changes over time in 3D images of tractography delineated by XTRACT. Changes over time in 3D images of tractography delineated by XTRACT. (A) Day 0. (B) Day two. (C) Day 42. With the transition of fractional anisotropy value, the visualization of tractography was increased, especially in the arcuate fasciculus (AF) (red arrowheads). Yellow areas: AF; blue areas: inferior fronto-occipital fasciculus (IFOF); green areas: inferior longitudinal fasciculus (IFL).

Based on these findings, we determined that the patient had CVST of the left superior sinus. Anticoagulation therapy with heparin was started immediately. Severe aphasia was resolved by the second day of treatment. Anticonvulsants were introduced after improving the severe sensory aphasia to prevent secondary epilepsy. MRV improved the left sigmoid sinus, and ASL showed no laterality. The FA values of white matter fibers related to language function in DTI were tracked using XTRACT, which performs auto-segmentation of probabilistic tractography [9]. The relationship with symptoms is shown in Figure 2.

After the resolution of severe sensory aphasia, FA values in the arcuate fasciculus (AF) decreased, while the patient continued to experience difficulty reading Chinese characters. As his dysfunction improved, FA values in the inferior fronto-occipital fasciculus (IFOF) and inferior longitudinal fasciculus (ILF) also decreased, likely reflecting a reduction in venous congestion (Table 1).

**Table 1:** The transition of fractional anisotropy values in each tractography. Fractional anisotropy value, mean ± standard deviation. AF: arcuate fasciculus; IFOF: inferior fronto-occipital fasciculus; ILF: inferior longitudinal fasciculus.

	Day 0	Day 2	Day 42
AF	0.32±0.15	0.31±0.14	0.31±0.11
IFOF	0.34±0.12	0.32±0.12	0.34±0.13
ILF	0.30±0.12	0.29±0.10	0.29±0.12

The patient was switched to direct oral anticoagulant therapy from heparin to prevent recurrence. His rehabilitation progressed smoothly, and he was discharged home.

MRI acquisition, imaging conditions, and image processing were conducted as follows. A 1.5 Tesla MRI system (SIGNA Explorer 1.5T, GE Healthcare Japan, Tokyo, Japan) equipped with an eight-channel coil was used. Imaging sequences included fluid-attenuated inversion recovery (repetition time (TR)/echo time (TE): 6.2/0.11 ms, slice thickness: 1 mm, matrix size: 512 × 512), diffusion weighted image (TR/TE: 4339/86 ms, slice thickness: 6 mm, matrix size: 256 × 256, b-value: 1000 s/mm²), MRV (TR/TE: 21/5.4 ms, slice thickness: 1.8 mm, matrix size: 512 × 512), ASL (TR/TE/post-labeling delay (PLD): 4843.0/10.5/2025 ms, slice thickness: 6 mm, matrix size: 512 × 512), and DTI (TR/TE: 12255/96 ms, slice thickness: 2.5 mm, matrix size: 256 × 256). DTI included 30 images with non-collinear diffusion gradients (b = 1000 s/mm²) and non-diffusion-weighted imaging (b = 0 s/mm²) scans. For image processing, probabilistic tractography was obtained using FMRIB software and MRtrix3 [10,11]. FA values of language-related tracts were calculated using the XTRACT tool in FSL (FMRIB Software Library) [9]. XTRACT is a probabilistic tractography method that automatically extracts major white matter pathways from diffusion MRI data, improving reproducibility through standardized spatial processing. This method processes data in standard space, allowing easier comparison between subjects and studies while extracting multiple pathways simultaneously. XTRACT calculates important quantitative values, including volume, FA, and mean diffusivity for each extracted pathway, offering flexibility for various research applications.

## Discussion

We evaluated the recovery process of the sensory aphasia due to CVST through ASL and DTI on MRI and found that DTI was particularly useful in the process of improvement of higher brain functions. Anticoagulation therapy facilitated thrombus regression over time [12]. Typical symptoms of CVST include headache, visual field disturbances, and focal neurologic symptoms, but this case did not present with typical edema on FLAIR imaging. The occlusion site of MRV was close to the transverse-sigmoid junction of the sigmoid sinus, which is the site of inflow of the vein of Labbe. This occlusion was thought to have caused congestion in the posterior and posterior temporal lobe, resulting in higher brain dysfunction, mainly sensory aphasia. During the acute phase of admission, MRV showed increased signal intensity in the parietal cortical vein, particularly in the vein of Trolard (Figure 1) [13]. Concurrently with the normalization of CBF laterality on ASL, MRV also showed improvement in the sigmoid sinus, and the signal asymmetry in the cortical veins resolved as well. In this study, we utilized XTRACT to evaluate each tract. XTRACT calculates the mean and standard deviation of FA values for the entire tract. While this approach may reduce the sensitivity to FA changes in focal lesion areas, it ensures methodological consistency by minimizing arbitrariness and improving reproducibility. Conversely, region-of-interest (ROI)-based methods may offer higher sensitivity to local FA changes, but they introduce challenges in subjectivity and reproducibility, even when anatomically guided. This represents a trade-off between sensitivity to lesion effects and measurement consistency. Since the present case exhibited multiple language symptoms rather than a focal lesion, we prioritized time-series reproducibility and opted for a whole-tract analysis using XTRACT. This approach allowed us to systematically track FA changes across all affected white matter pathways without the variability introduced by ROI selection [14]. Furthermore, Mochizuki et al. also employed XTRACT for whole-tract FA evaluation in a similar context, supporting the validity of this approach [8]. Their study highlights the advantage of automated tractography in minimizing user bias while providing reproducible measurements across subjects. As this is a single-case study, inferential statistical analysis was not conducted. However, we analyzed temporal trends in FA values to assess white matter remodeling during recovery. Whereas prior studies have reported long-term FA increases as symptoms improve [15]. This discrepancy may be attributed to the shorter observation period in our study (a few days to 40 days), as FA values have been shown to fluctuate dynamically in the early phases of neuroplasticity. Given that similar temporary FA reductions have been documented during symptom recovery, we believe that our study captures this transient phase of white matter remodeling [8,14]. While 3.0 T MRI generally offers a higher signal-to-noise ratio, Moser et al. reported no statistically significant difference in FA values between 1.5 T and 3.0 T MRI [15]. Given this, the use of a 1.5 T system in our study is unlikely to have significantly impacted FA measurements. Therefore, the effect of using a 1.5 T MRI on FA values appears to be minimal or limited [15]. As the FA values tracked the transition of aphasia symptoms in stroke, it was inferred that the gradual improvement of each fiber reflected the function of each white matter fiber [6,7]. It would be useful to evaluate the transition of FA as the functional recovery progresses during the process of improvement of cerebral blood flow congestion associated with CSVT. In this case, the finding in ASL was an increase in CBF with observation at the time when the symptoms of sensory aphasia were most intense. Previous studies have reported that in cases of complete venous occlusion, CBF decreases due to congestion, accompanied by an increase in cerebral blood volume [5,16]. Furuya et al. reported cases where FLAIR showed edema, and seizures were present, but CBF did not increase [5]. In contrast, our case demonstrates that ASL may show increased CBF even when FLAIR does not show edema, assuming that epileptic seizures are not present. This result was contrary to our results. The fact that our case was not completely occluded may have contributed to the increased CBF. Although seizures cause increased CBF in ASL, we did not observe any symptoms suggestive of lesion-related seizures during the study period. In any case, the changes in findings in ASL reflect changes in CBF during the acute phase of the disease and were useful in assessing the course of the disease. Our results suggest that the combination of MRI ASL and DTI is useful in the evaluation of CVST and higher brain dysfunction. A limitation of this report is that it is a single case, and statistical analysis was not possible. However, it is possible to obtain an imaging index of functional improvement by tracking the improvement of language function and the transition of FA values in each tract and to evaluate the progression of the disease using ASL. Even in the rare disease of CVST, it may be possible to quantitatively evaluate the pathological transition of stroke, as previously reported.

## Conclusions

In this case, we used ASL to assess CBF alterations associated with CVST. Additionally, we used DTI to evaluate the recovery of language-related dysfunction, including sensory aphasia caused by venous congestion. A trend was observed between sensory aphasia and FA values in the AF, reflecting structural changes during recovery. Furthermore, the recovery of logographic language processing, including Chinese character reading, appeared to correspond with changes in the IFOF and ILF. These findings suggest that ASL and DTI may be valuable tools for assessing disease progression and monitoring recovery in CVST-related higher brain dysfunction.
